# Medical student reporting of factors affecting pre-clerkship changes in empathy: a qualitative study

**Published:** 2013-03-31

**Authors:** Hasan Sheikh, Jennifer Carpenter, Joy Wee

**Affiliations:** 1 Family Medicine Resident, University of Toronto, Toronto, Ontario, Canada; 2 Department of Emergency Medicine, Queen’s University, Kingston, Ontario, Canada; 3 Department of Physiatry, Queen’s University, Kingston, Ontario, Canada

## Abstract

**Objective:**

To isolate factors that medical students identify as possibly affecting empathy in pre-clerkship years of medical school.

**Methods:**

12 students in their second year of medical school at Queen’s University were randomly selected and asked to participate in semi-structured interviews conducted from an ethnographic perspective.

**Results:**

Students reported both negative and positive changes in empathy. Negative changes included desensitization and focusing on the disease process, decreased ability to see things from patients’ perspectives, and routine responses in emotional situations. These changes occur due to time constraints, objective lessons in empathy, and a changing identity. Positive changes included an increased awareness of the impact of illness, and increased ability to read feelings. These changes result from increased exposure to patients, discussions surrounding the psychosocial impact of illness, and positive role models.

**Conclusion:**

Students should be made aware of the limitations of objective lessons in empathy, and non-evaluated, implicit lessons should be emphasized when possible. Students should be encouraged to maintain relationships outside of medicine. Aspects of medical school that currently promote empathy should be reinforced, including exposure to patients, opportunities to work closely with positive role models, and practical discussions surrounding the psychosocial impact of illness.

## Introduction

Empathy is a poorly defined concept about which there is little consensus in the literature. It has been described as the cognitive process of taking another’s perspective,^1^ while at other times it has been described as including emotional reactions such as feelings of warmth and sympathy towards others.^2^,^3^ A number of studies have been done to evaluate changes in medical students’ empathy as they progress through their years of study, and results have similarly lacked consensus.

Some studies conclude that medical students’ ability to be empathetic declines throughout medical school. Attitudes change, even in the pre-clerkship years, when students’ exposure to patients is limited.^4^ It was suggested that student’s attitudes towards social factors and paramedical co-operation, in particular, become more negative during those pre-clerkship years.^4^ Students’ emotional intelligence, defined as the “verbal and nonverbal appraisal and expression of emotion, the regulation of emotion in the self and others, and utilization of emotional content in problem solving,”^5^ was shown to decrease, even in pre-clerkship years.^3^ In particular, Attention to Feelings (the extent to which one attends to the verbal and nonverbal expression of emotion), Empathetic Concern (feelings of concern, warmth, and sympathy towards others), and Mood Repair (the ability to moderate unpleasant moods and maintain pleasant ones) have been reported to significantly decline throughout the first four years of medical training.^6^ Vicarious empathy, defined as the response to perceived emotional experiences of others, similar to the concept of Empathetic Concern used in other studies, has been shown to decrease over the four years, including the pre-clerkship years.^7^

However, other studies have provided evidence to the contrary, citing that the change in empathy is limited to the transition from pre-clerkship to clerkship, related temporally with increased exposure to patients and increased responsibilities.^8^,^9^ At most schools in Canada and the United States, this transition occurs between the second and third year. Yet other studies have concluded that medical students become more empathetic throughout medical school.^10^ The ability to manage and regulate emotions was shown in one study to be greater in students who were at the end of their training compared to those at the beginning of their training.^10^ The significance of any of these findings has been disputed.^11^

The discussion about empathy continues in the literature. We believe this is for two reasons: plausibility and importance. Those who have gone through medical school can see the possibility that medical school changes students’ attitudes, personalities, and reactions to emotional situations. We also believe that most intuitively think that empathy is an important quality to have in clinical practice. Indeed, its value has been shown to be in the literature, correlating to better medical management, such as higher control of HbA1c targets in diabetics, and LDL-C levels.^12^

The factors behind possible changes in empathy, either positive or negative, have been explored in a variety of ways in the literature. Open-ended surveys, with highly variable response rates, have suggested that poor role models, overly demanding patients, lack of appreciation, fear of malpractice, fear of making mistakes, a demanding curriculum, time pressure, sleep loss, and hostile environments contribute to a decrease in empathy.^9^,^13^ Some involved in medical education have likened such features to being part of a neglectful and abusive family system, with unrealistic expectations, denial, indirect patterns of communication, rigidity, and isolation.^14^ To our knowledge, however, no study has been done that explores students’ views on the factors affecting their attitudinal changes, personality changes, or changes in their ability to be empathetic, through in-depth interviews. This pilot study explores such changes from the students’ perspectives, in the hopes of elucidating factors that both positively and negatively influence empathy, in order to identify which aspects of medical school are helpful in developing and maintaining empathy, and which ones are detrimental.

## Methods

### Participants

12 randomly selected second year medical students’ perceptions were explored using semi structured interviews from an ethnographic perspective. They were selected using a random number generator and an alphabetical list of the class. One student declined to be interviewed. Each student who agreed to be interviewed signed consent forms prior to the interview, and ethics approval was obtained by the Research Ethics Board at Queen’s University.

### Procedures

Interviews lasted between one and two hours. An interview guide with prompts was developed based on the literature search and informal discussions with students. Topics discussed included, but were not limited to: factors influencing empathy and personal development, inspirational examples during medical school, examples of cynicism during medical school, professionalism, analyses of each type of learning environment (including didactic lectures, team-based learning, problem-based learning, ethics class, clinical skills, and OSCEs), identity as a medical student compared with one’s identity outside of medical school, and the social environment within medical school.

#### Assumptions

The idea for this study arose from discussions in the first year of medical school on the loss of empathy as evidenced by research in the literature. It was therefore in this context, and the further exploration of the literature, that the study was conducted. There was an assumption that empathy decreases throughout medical school, and it was in that light that students were asked to analyze the factors that cause that change, as well as any factors that might help preserve their empathy or insulate against its loss.

#### Settings and Students

This study was conducted at Queen’s Medical School in Kingston, Ontario, Canada. The Queen’s undergraduate medical curriculum lasts four years. Students must have already completed at least three years of an undergraduate degree before entering the undergraduate medical program. Students come from a wide range of backgrounds. At the time of the interviews, the first two and a half years at Queen’s were pre-clerkship years and consisted of a combination of lectures, team-based learning sessions, problem-based learning sessions, and a clinical skills sessions that involved patient contact roughly once a week. All students participating in this study were in their second year of their degree. They were asked to focus on factors affecting pre-clerkship empathy, compassion, and personality development.

#### Analysis

Each interview was tape-recorded and transcribed verbatim. Each transcript was analyzed for ideas reflecting the students’ *beliefs, perceptions of their training, feelings, actions that they had taken,* and *personal anecdotes*. These were further subcategorized into discussions on: *explicit lessons in empathy vs. experiential lessons, focusing on the disease process, the nature of empathy, desensitization, identity, patients as learning tools, personality changes, positive role models, professionalism, time, feeling inspired, feeling cynical,* and *the different learning environments in medical school*. These themes were then analyzed for recurrent patterns between students and conclusions were drawn. These conclusions were independently verified by the two co-authors on three transcripts each.

## Results

### The Nature of Empathy

Students found that self-reporting changes in empathy and personality was extremely difficult:

“It’s hard to really think back to the start of medical school and think about what my personality was and now how it’s changed, because it’s...like...when someone’s lost weight over a period of time, I don’t notice it changing, but then someone I haven’t seen in two years will come back and say ‘oh you lost weight’ and it’s like ‘really, I guess I have, but I haven’t noticed it’.

Students were, however, able to comment on factors that may positively and negatively influence their ability to be empathetic. And while many students believed that how they practiced clinically during their training would be very different from how they would practice in the future, an idea arose in the discussions about how empathy acts as a positive feedback cycle:

“If I’m empathetic towards [the people around me], they’re going to talk to me more. And we’ll just have a better relationships. So I think people around me...encourage that.”

### Negative influences on empathy

Students identified a few domains in which empathy seemed to decrease. Students found that they were becoming desensitized to suffering, and they often found themselves focusing primarily on the disease process:

“I’d drive by and there’s a car crash - my first instinct would be...where is the patient, [and] what injuries do they have. And that’s really horrible to notice about yourself.”

“We do tend to begin to block out [processing the emotions related to seeing those who are suffering]”

“I notice ‘Oh I’m thinking too hard about the actual condition itself’”

Students did notice that this focus on the disease process resulted in a seeming decrease in empathy:

“[Focusing on hard data] does...take away attention from [the feelings of the patient]. If I only have a limited attention span then, it does seem like it would take away from viewing them as a person with feelings.”

Students reported a decreased ability to see things from patients’ perspectives as they progressed through medical school, also leading to desensitization and a loss of empathy:

“I wouldn’t be able to feel the exact same way or understand what [patients with a new diagnosis] are going through - like for them, they’ve never even heard of this illness, but for me since I’ve been exposed to it I know that people can get this condition.”

Additionally, students found that, throughout the process of their training, they began to have routine responses to emotional situations, even outside of medical school and clinical skills training:

“My aunt passed away of cancer last month, and, I didn’t know her very well - and so, when they first told me they had pancreatic cancer - I went right into well what are her symptoms - that’s not the ideal response you would like to respond to a family member having cancer.”

Students reported that a lack of time to process their emotions contributed to desensitization:

“I think that in medical school we see a lot of images and we hear a lot of stories, of things that are, that could be considered very, very stressing. And we don’t want to spend time dealing with these issues, we have other things to spend our time with, for example studying. So we don’t want to spend time battling with our own emotions, so we do tend to begin to block out a lot of these things.”

This lack of time, combined with “objective lessons” in empathy, particularly in clinical skills, resulted in these objectives being completed as efficiently and superficially as possible. “Objective lessons” were defined in the discussions as situations in which students were expected to demonstrate their empathetic nature as part of an evaluation. Such situations included clinical skills sessions, in which students were evaluated for their ability to explore the feelings of standardized patients, and Problem-Based Learning cases, when students were asked to discuss the psychosocial context of a hypothetical patient’s illness.

“Because you’re forced to complete all these things within a time limit and you’re worried about doing everything accurately - I think that maybe that’s why it becomes mechanical.”

The combination of lack of time and objective lessons in empathy can also contribute to the desensitization process, as these lessons are sometimes mocked by students or may seem farcical.

“Some of my friends mock [ethics class when issues of empathy come up], because it seems like they’re trying to teach you something that inherently can’t really be taught.”

“[Clinical skills] had just begun to feel like too much of a game...[it] made it very hard to take things seriously.”

“I think that probably the way that they write the [PBL] cases are a little bit cheesy, and sometimes because it’s so cheesy, a lot of what would have been considered the human factor probably is laughed off.”

Students also reported that their identity, their view of themselves and how they believed others perceive them, changed throughout the pre-clerkship phase of medical school. Students found that growth in their identity as medical students was accompanied by a loss of their identity outside of medical school:

“My identity outside of medical school - that’s very difficult because I don’t have much of one.”

This identity narrows as a result of a few factors. First, the limited amount of time spent outside of medical school and the large amount of time spent with other medical students who have similar levels of education and similar knowledge bases.

“Med School, by virtue of the time constraint and the people constraint, has limited my horizons quite a bit.”

Second, students begin to form a camaraderie amongst medical students, as they begin to feel that those outside of medicine do not understand the stresses they encounter.

“We are going through a war together...we are forming the sort of connections that you form when you go through a war together. And I wonder how close any of us will be, myself included, to people outside of medicine at the end.”

Finally, students’ desire to learn as much as possible, with the view that that knowledge will aid them in helping patients in the future, can cause them to spend their limited free time on medically-related endeavors, resulting in further narrowing of their identity:

“Medicine is an endless sea...and in the future everything we do that is not medicine is going to be things taking away from medicine.”

The result of this narrowing of one’s identity is a decreased ability to put one’s self in the patient’s shoes, to see things from the patient’s perspective.

“When you are...interacting only with medical professionals, you start to have your opinions of what is norm in a person...altered. Because as much as there are very inherent differences in all of our personalities, we are all smart, we are all well educated, we are all very driven, relatively very professional individuals. We’re able to handle bad news, so when you start to see that as normal, there becomes a risk that you will associate that ability with everyone and you will assume that everyone can do that kind of thing. [You assume that] they can understand what you’re saying, they can deal with fact that you’re talking about something bad and not crying about it.”

Students found that maintaining an identity outside of medicine had the opposite effect. Maintaining relationships outside of medicine helped insulate against the loss of empathy; in particular, it helps keep a perspective on the stresses of medical school, aid in the prevention of desensitization, improve one’s ability to consider the patient’s perspective, and remember the importance of compassion and empathy.

“The people outside of medicine, certainly they give me that much different perspective I don’t get in medicine....One, how lucky I am to be in medicine...it’s one of the greatest privileges that can be bestowed upon anyone....Another thing is the fact that the problems that I have in medicine aren’t real problems.”

“We shouldn’t get used to suffering, we shouldn’t adapt to it, and say that’s the norm....If you spend a lot of time with people who are suffering you should spend time with people who aren’t.”

“[When] you find yourself explaining stuff to [family and friends outside of medicine]...[you] realize that these things are foreign concepts to people. Not everyone deals with hearing news like this the same way.”

“Having relationships with people who are outside medicine has been very important for me to remember what is important, to remember the big picture, to remember the patient and the empathy and those kinds of interactions.”

### Positive influences on empathy

Students reported factors in medical school that increased their ability to be empathetic. They began to have an increased awareness of the impact of illnesses and the signs of illness.

“I now see things that I didn’t see before...I think I’ve come to notice a lot of things that once a upon a time I would have ignored....It may have made me more [empathetic], because things that would have once been noise are now a signal to me and are now significant.”

Some students also found that their skills improved in certain domains of the clinical encounter, namely, the ability to read feelings and to listen:

“[My ability to be empathetic is] about the same, to maybe even a bit improved in reading feelings.”

“Medical school has been beneficial in showing me how to listen to my friends and how to just be there for them.”

This positive change in empathy occurs for several reasons. First, students are exposed to patients regularly throughout medical school, and this increased exposure to patients allows them to exercise their empathy, maintaining the positive feedback loop discussed earlier.

“Spending some time in the hospitals and seeing patients...interacting with an extra variety of people.”

In addition, learning about the psychosocial impact of illness has resulted in an increased knowledge about the patient experience, resulting in a greater ability to put oneself in the patients shoes, and to see things from patients’ perspectives:

“It’s allowed me to appreciate more the difficult struggle that is associated with some of these medical conditions I may have previously in a juvenile immature way almost made fun of. Or not understood the extent of previously. So I would say that I’m probably more compassionate now that my knowledge has increased.”

“When they bring in a patient, I think that would probably increase my compassion and empathy....Last semester we had this session with patients that had spinal cord injuries. For me that increased my empathy to see how their lives were and to...they talked about what they’re able to do and what they’re not able to do and everything from personal perspective to me - that increased my awareness and desire to learn more about them.”

Additionally, positive role models remind students about the positive impact physicians can have in a patient’s life and the enjoyment physicians can take from their job. This not only puts into perspective the stresses of medical school, thereby diminishing them, but can also feed into the positive feedback cycle of empathy:

“Observerships I’ve done really allowed me to see how much [some physicians] enjoy their job, and how much they love helping people.”

“My impression would be that it’s positive exposure, positive role models, [that highlights the importance of empathy].”

## Discussion

This is a relatively small study that allowed students to discuss both negative and positive factors that influenced empathy, and these factors will need to be explored further in future studies with a larger and more varied sample. Nonetheless, some interesting discussions arose. The idea of empathy as a positive feedback cycle came up in interviews with various medical students. If it is indeed true, and to the best of our knowledge there is no literature exploring the development or maintenance of empathy in adulthood, it would then stand to reason that a temporary decline or increase in empathy throughout medical school carries the risk/opportunity of translating into students’ empathetic abilities as practicing physicians.

With respect to negative changes in empathy, lack of time was a factor that was often brought up by the participants. Medicine, however, is a never-ending study, and a drastic decrease in the amount of hours spent learning would be unrealistic and likely negatively impact quality of care. The lack of time interacts with objective lessons in empathy to cause superficial and efficient completion of objectives that relate to empathy. It is important that the limitations of these objective lessons be made explicit to students, and should be balanced with implicit lessons in empathy, whereby students spend time with patients, to explore the psychosocial context of the patient’s illness in a non-evaluated, non objective-based way. This may not only help insulate against the risk of objective lessons becoming farcical to students, but may aid students in their ability to see illness from the patients’ perspectives. One such intervention could be for students to follow a patient in the community longitudinally over the pre-clerkship years, accompanying them to their medical appointments. The experience of hearing their stories and seeing how they navigate through the healthcare system would provide implicit lessons in the impact of illness on the patient, without students feeling the need to superficially satisfy specific empathy-related objectives.

Each interview also included a lengthy discussion about the concept of identity as a medical student, one’s identity outside of medicine, and how these may relate to changes in empathy. Identity formation is an ongoing and self-directed process, one that occurs in stages related to self-exploration and personal commitment^15^,^16^,^17^. The process of identity formation involves changes in one’s self-perception, beliefs, values, and perspectives.^18^,^19^ There is little research into formation of a professional identity amongst medical students, but one study from the University of Helsinki found that medical students rapidly developed an identity as a future doctor in their first year of clinical contact.^20^ Another study found that students in their preclinical years were fairly evenly distributed between the various stages of commitment to their new professional identity.^21^ To the best of our knowledge, there is no literature on the impact of identification as a medical student or future physicians on empathy or compassion. In our study, students reported that spending the majority of one’s day with individuals who are all similarly well educated, with similar knowledge bases, carries the risk of shifting a student’s perspective of the average individual.

The small sample size of our study prevents the drawing of definitive conclusions, but the results suggest that students may begin to lose sight of how the ‘typical individual’ might understand illness, and individual ability to cope with bad news about health. This can be countered by encouraging students to maintain relationships outside of medicine and highlighting the benefits of this with respect to their ability to be empathetic. This may also mitigate some of the desensitization that may arise from being around illness all day. Such interactions may provide illness-free time that can help students better appreciate the novelty of illness to patients. It is interesting that students’ desire to acquire as much knowledge as possible, in order to benefit their future patients, can lead to this increased identification as a medical student, and paradoxically lead to a loss of empathy. Therefore, it is conceivable that encouraging students to maintain these relationships in the context of a changing identity and the risks that carries may be effective in preventing some of these losses. Larger studies will have to be done that focus in greater detail on the formation of identity in medical school and how it interacts with changes in empathy and personality to supplement our preliminary results.

Fortunately, students identified factors in medical school that promoted their ability to be empathetic: learning more about illness, from the signs of specific illnesses to the impact that these illnesses have on patients’ lives, appears to contribute to a heightened awareness for students and improve their ability to be empathetic. Exposure to patients provides opportunity for experiential learning and practice of skills related to empathy: asking the right questions, picking up on patients’ body language, and demonstrating empathy. Students also felt that positive role models significantly impacted their development of empathy, as they highlighted the importance of empathy in the clinical encounter, and inspired students to act in an empathetic way, thereby feeding into the positive feedback cycle of empathy.

There were a few limitations to this study. Based on the literature and discussions in the first year of medical school, there was a pre-existing bias that empathy was negatively influenced throughout medical school, and therefore, the discussions focused more on negative influences than positive influences. In addition, only 12 participants were interviewed, and all from one class and from one medical school. Given the differences between the curriculums of various medical schools and the differences between the culture of medicine in various countries, these results may not be representative of all medical students. Therefore, the results of this study must be verified with a larger sample size and in different environments.

### Conclusion

Students identified both positive and negative influences on empathy in their pre-clerkship years of medical school, even if they were unable to comment on an overall change. Negative influences included a lack of time to process one’s emotions, objective lessons in empathy that are superficially completed, and a change in one’s identity. Such influences may lead to desensitization, the formation of routine responses in emotional situations, and a decreased ability to see things from patients’ perspectives. Positive influences included increased exposure to individuals with illnesses, discussions surrounding the psychosocial impact of illness in a non-evaluated way, and positive role models. These were thought to foster an increased awareness of the impact of illness on an individual’s life, and an increased ability to read others’ feelings. More in-depth research into factors influencing empathy in medical school is needed.
